# In Vitro Screening and Lipid-Lowering Effect of Prickly Pear (*Opuntia Ficus-Indica* L. Mill.) Fruit Extracts in 3T3-L1 Pre-Adipocytes and Mature Adipocytes

**DOI:** 10.1007/s11130-023-01137-8

**Published:** 2024-01-11

**Authors:** Itziar Eseberri, Andrea Gómez-Maqueo, Jenifer Trepiana, Iván Gómez-López, Carina Proença, M. Pilar Cano, Maria P. Portillo

**Affiliations:** 1 Nutrition and Obesity Group, Department of Nutrition and Food Science, University of the Basque Country (UPV/EHU) and Lucio Lascaray Research Institute, Vitoria, Spain; 2 Bioaraba Health Research Institute, Vitoria, Spain; 3 CIBERobn Physiopathology of Obesity and Nutrition, Institute of Health Carlos III, Madrid, Spain; 4 Department of Biotechnology and Microbiology of Food, Institute of Food Science Research (CIAL, CSIC-UAM), Nicolás Cabrera 9, Madrid, 28049 Spain; 5 REQUIMTE, Laboratory of Applied Chemistry, Department of Chemical Sciences, Faculty of Pharmacy, LAQV, University of Porto, Porto, Portugal

**Keywords:** *Opuntia ficus-indica*, Prickly pear, 3T3-L1 adipocytes, Betalains, Piscidic acid, Isorhamnetin glycosides

## Abstract

*Opuntia ficus-indica* fruits have been widely used due to their nutritional composition and beneficial effects on health, particularly against chronic diseases such as diabetes, obesity, cardiovascular diseases and cancer, among others. In recent years, prickly pear peel and pulp extracts have been characterised, and a high number of bioactive compounds have been identified. This study aimed to analyse the triglyceride-lowering effect of prickly pear peel and pulp extracts obtained from fruits of three varieties (Pelota, Sanguinos, and Colorada) in 3T3-L1 maturing and mature adipocytes. At a concentration of 50 µg/mL, peel extracts from Colorada reduced triglyceride accumulation in pre-adipocytes and mature adipocytes. Additionally, at 25 µg/mL, Pelota peel extract decreased triglyceride content in mature adipocytes. Moreover, maturing pre-adipocytes treated with 50 and 25 µg/mL of Sanguinos pulp extract showed a reduction of triglyceride accumulation. In addition, the lipid-lowering effect of the main individual betalain and phenolic compounds standards were assayed. Piscidic acid and isorhamnetin glycoside (IG2), found in Colorada peel extract, were identified as the bioactive compounds that could contribute more notably to the triglyceride-lowering effect of the extract. Thus, the betalain and phenolic-rich extracts from *Opuntia ficus indica* fruits may serve as an effective tool in obesity management.

**Supplementary Information:**

The online version contains supplementary material available at 10.1007/s11130-023-01137-8.

## Introduction

Obesity is recognised as one of the most alarming public health problems. According to the World Health Organization (WHO), in 2022 more than 1 billion people worldwide suffered from obesity, and this number continues to increase each year [1]. In addition to being a disease on its own, obesity increases mortality as it is a risk factor for the development of several co-morbidities, including type 2 diabetes, cardiovascular diseases and some types of cancer, among others [2]. Obesity is defined as excessive fat accumulation, caused by changes in adipose mass. Adipose tissue development is determined by both hyperplasia (increase in adipocyte number) and hypertrophy (increase in adipocyte size). In this context, adipogenesis is the cellular differentiation process involved in adipose tissue hyperplasia, in which the fibroblast-like progenitor cells turn into mature adipocytes. Although the adipocyte count can change in adulthood, the modification of adipocyte size is the main mechanism for adulthood fat mass fluctuation [3, 4].

*Opuntia spp.* belongs to the Cactaceae family, a group that comprises more than 300 species native to the American continent. *Opuntia* grows in very adverse conditions, making it especially interesting for cultivation in arid regions around the World [5]. It has been used for centuries both as a food source and in traditional folk medicine, owing to its nutritional properties and associated health benefits, particularly in addressing chronic diseases such as diabetes, obesity, cardiovascular diseases and cancer [6]. *Opuntia ficus-indica* L. Mill. is the most widely consumed species due to its tasty cladodes and fruits. Its popularity extends to countries like Mexico, Spain, Italy, Morocco, Argentina and Chile.

Prickly pear fruits have been widely characterised in the last few years and are rich in several bioactive compounds, such as betalains, piscidic acid, isorhamnetin glycosides (IG´s), ascorbic acid and fibre, among others [7–10]. Although there is no data in relation to prickly pear extracts, there is scarce information regarding the anti-obesity effects of other *Opuntia* products. In a study devoted to analyse the effect of three fruit vinegars in obesity-induced cardiomyopathy, the authors observed that the treatment with prickly pear vinegar prevented the increase on body weight and plasma inflammatory parameters in Wistar rats fed a high-fat diet [11]. In addition, Verón and co-workers stated that prickly pear juice fermented with *Lactobacillus plantarum* S-811 was able to decrease body weight and obesity-associated insulin resistance in obese mice [12]. With regard to bioactive compounds, the impact of isorhamnetin and its derivatives on adipogenesis and triglyceride accumulation in cultured adipocytes has been previously studied [13]. In the case of betalains, only one study has reported the anti-adipogenic effect of betanin in 3T3-L1 preadipocytes [14]. To our knowledge, no studies focusing on the potential anti-obesity effects of piscidic acid have been published.

**Fig. 1:**
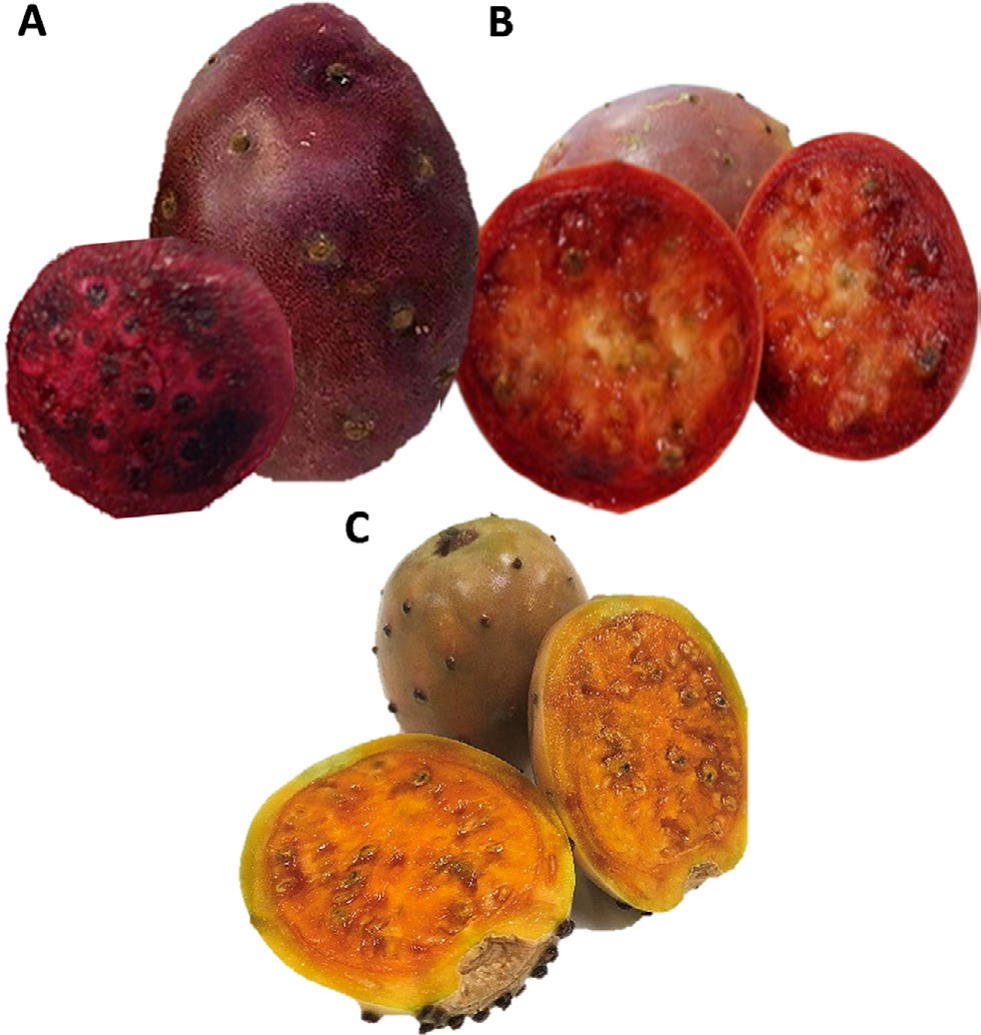
Pelota (**A**), Sanguinos (**B**) and Colorada (**C**) prickly pear varieties

There is significant interest in comparing the biological responses among different *Opuntia* varieties, given the notable differences in betalain and polyphenol content and profiles. In fact, the composition of betalains is the reason for having red, green, purple, yellow, orange and white coloured fruits, with betaxanthins contributing yellow-orange hues and betacyanins providing red-violet tones [6]. For the present study, we selected three widely cultivated varieties of *Opuntia*: a Mexican violet variety (Pelota), and two Spanish types, one red (Sanguinos) and one orange (Colorada), to analyse and compare their composition and potential bioactivity (Fig. 1).

Taking into account the great interest in *Opuntia*, and the limited information about the anti-obesity effects of prickly pear extracts, the present work aimed to study the potential lipid-lowering effects of *Opuntia ficus-indica* L. Mill. fruit peel and pulp extracts, obtained from three Mexican or Spanish varieties (Pelota, Sanguinos and Colorada), in 3T3-L1 pre-adipocytes and mature adipocytes, to select the most interesting one for a future in vivo study. Furthermore, the contribution of the main individual bioactive compounds (betalains and phenolic compounds) of the selected extract to its lipid-lowering effect was analysed. We hypothesised that the differences in bioactive compound composition among the three varieties of *Opuntia ficus-indica* are linked to variations in lipid-lowering effectiveness.

## Materials and Methods

The materials and methods section is presented as an Online Resource.

## Results and Discussion

### Extract Composition

Table 1 shows the main betalains and phenolic compounds present in the six fruit extracts. With respect to betalains, indicaxanthin was found in higher amounts in Colorada prickly pear, while betanin was predominantly present in Pelota and Sanguinos varieties. In all three, betalains were more abundant in the fruit pulp than in the peel. Concerning the phenolic compound piscidic acid, it was primarily present in fruit peels. Upon comparison, Pelota fruit peel exhibited the highest amount of this phenolic acid, although the levels were quite similar in all three varieties. Lastly, IGs were exclusively present in the peel of the studied prickly pear fruit varieties. The amounts of IGs were quite similar in all three, except in Pelota peel extract, which contained lower amounts of IG2 and IG4.

**Table 1:** Quantification (µg/g dry extract) of betalains and phenolic compounds in prickly pear (*Opuntia ficus-indica* L. Mill.) tissue extracts

*Opuntia ficus indica* L. Mill. extracts						
	Pelota		Sanguinos		Colorada	
Compound	Peel	Pulp	Peel	Pulp	Peel	Pulp
Indicaxanthin	0.06 ± 0.00	0.23 ± 0.01	0.09 ± 0.00	0.12 ± 0.01	0.45 ± 0.02	0.74 ± 0.04
Betanin	1.88 ± 0.09	2.79 ± 0.14	0.80 ± 0.04	0.27 ± 0.01	0.09 ± 0.00	0.08 ± 0.00
Piscidic acid	68.79 ± 3.44	4.19 ± 0.21	52.56 ± 2.63	6.46 ± 0.32	50.99 ± 2.55	5.79 ± 0.29
IG1^1^	0.59 ± 0.03	n.d.	0.34 ± 0.02	n.d.	0.22 ± 0.01	n.d.
IG2^2^	0.10 ± 0.03	n.d.	0.29 ± 0.01	n.d.	0.21 ± 0.01	n.d.
IG4^3^	0.05 ± 0.00	n.d.	0.22 ± 0.01	n.d.	0.12 ± 0.01	n.d.
IG5^4^	0.38 ± 0.02	n.d.	0.46 ± 0.02	n.d.	0.32 ± 0.02	n.d.

Data are means ± SEM (standard error of the mean) of three independent experiments carried out in sextuplicates. ^1^isorhamnetin glycosyl- rhamnosyl-rhamnoside (IG1), ^2^isorhamnetin glucosyl-rhamnosyl-pentoside (IG2), ^3^isorhamnetin glucosyl-pentoside (IG4), ^4^isorhamnetin glucosyl-rhamnoside (IG5). n.d.: not detected

Thus, the chromatographic analysis revealed that the phytochemical profile of *Opuntia ficus indica* depends on the variety and the fruit tissue (peel or pulp). The presence of higher amounts of piscidic acid in the peel extracts compared to pulp extracts align with findings from a previous study in our lab, where the peels of other *Opuntia ficus-indica* L. Mill. varieties (Fresa, Blanco Buenavista and Blanco Fasnia) also exhibited greater amounts (10-fold) of piscidic acid compared to the pulps [9]. Moreover, the distribution of IGs found in the present study is in good accordance with that observed in other *Opuntia ficus-indica* L. Mill. varieties [9]. With regard to betalains, the extract of the Colorada variety, characterised by its orange colour, contains a higher indicaxanthin content compared to the other two varieties. This observation aligns with findings reported by Koss-Mikołajczyk et al. [15], where the authors noted a higher amount of betanin in the red variety, which is consistent with the findings of the present study [15].

### Effects of Prickly Pear Extracts on Triglyceride Accumulation and Cell Viability in Maturing 3T3-L1 Pre-Adipocytes

**Fig. 2:**
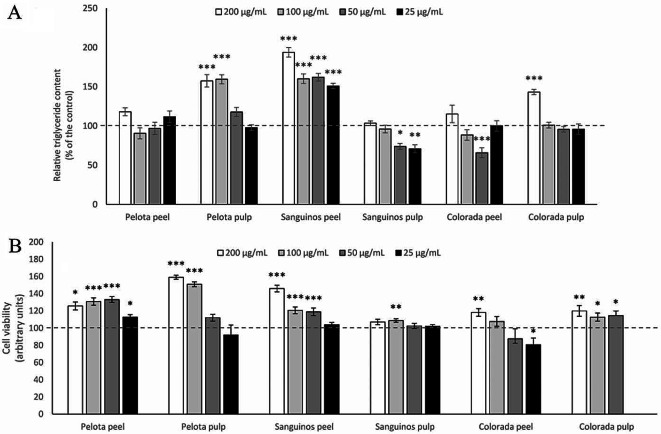
Triglyceride accumulation (**A**) and cell viability (**B**) in 3T3-L1 maturing pre-adipocytes treated from day 0 to day 8 of differentiation with 200, 100, 50 or 25 µg/mL extracts of Pelota, Sanguinos and Colorada *Opuntia ficus-indica* (L.) Mill. pulps and peels. Data are mean ± SEM (standard error of the mean) of three independent experiments. The intermittent line represents control values in pre-adipocytes. Student’s *t* test was used for the analysis of comparisons between each group of treated cells and the control group (*P < 0.05; **P < 0.01; ***P < 0.001)

The six prickly pear extracts were used for cell treatments at 200, 100, 50 or 25 µg/mL from day 0 to day 8 of differentiation (Fig. 2A). Some of the extracts significantly elevated triglyceride content in pre-adipocytes: Specifically, Pelota pulp extract resulted in increases of 57% and 59% at concentrations of 200 and 100 µg/mL, respectively; Sanguinos peel exhibited a rise of 93%, 60%, 62% and 51% at 200, 100, 50 and 25 µg/mL, respectively; and Colorada pulp extract led to a boost of 43% at the highest concentration (200 µg/mL). By contrast, concentrations of 50 and 25 µg/mL of Sanguinos pulp extract and 50 µg/mL of Colorada peel extract significantly reduced triglyceride accumulation in cells (-29%, -26% and -34%, respectively). Ultimately, none of the tested doses of Pelota peel extract elicited any effect (Fig. 2A).

On the other hand, to discard potential cytotoxic effects, cell viability was measured following treatment with the four doses of the six prickly pear extracts. In cells treated during the differentiation process, only 25 µg/mL of Colorada peel extract reduced cell viability (Fig. 2B). In contrast, several extracts at different concentrations significantly increased cell viability. For the Pelota variety, these included all the concentrations of the peel extract and 200 and 100 µg/mL of the pulp extract. The Sanguinos peel extract showed increases at 200, 100 and 50 µg/mL, with a tendency at 200 and 100 µg/mL for the Sanguinos pulp extract. The Colorada peel extract at 200 µg/mL and the Colorada pulp extract at 200, 100 and 50 µg/mL also exhibited increases. For Pelota pulp at 50 and 25 µg/mL, Sanguinos peel at 50 and 25 µg/mL, Sanguinos pulp at 50 and 25 µg/mL, Colorada peel at 100 and 50 µg/mL and Colorada pulp at 25 µg/mL, no alterations in cell viability were observed.

Considering that bioactive compounds are responsible for the biological effects of plant extracts, the variations in betalains and phenolic compound profiles identified among the six studied prickly pear fruit extracts could potentially lead to different lipid-lowering effects. This is the reason pre-adipocytes were incubated with four doses of the extracts. After the treatment of maturing pre-adipocytes, it was observed that Pelota pulp extract was ineffective. Among the other five extracts, some showed a pro-adipogenic effect (Pelota pulp, Sanguinos peel and Colorada pulp), while others demonstrated an anti-adipogenic effect (Sanguinos pulp and Colorada peel), as indicated by changes in cell viability and/or cell triglyceride content (Fig. 2).

The observed pro-adipogenic effect may be unexpected; however, considering that mono- or di-saccharides naturally present in the prickly pear tissues can be co-extracted in low amounts with betalains and phenolic compounds by the employed extraction solvent (methanol:water, 50:50, v/v), it is plausible that they may be present in the extracts. Consequently, their contribution to the observed triglycerides increase cannot be discarded [16]. In fact, Krishna et al. [17] studied the influence of monosaccharide and disaccharide concentrations (glucose, galactose, lactose and sucrose) added to 3T3-L1 pre-adipocyte differentiation media on their utilization as an energy source by cells during their differentiation process. Their findings revealed that both glucose and sucrose enhanced the adipogenic process [17]. On the other hand, it should be noted that, in line with the present results, other authors have also reported the pro-adipogenic effect of *Opuntia* or other plant extracts rich in bioactive compounds such as isorhamnetin [18]. In a study analysing the effect of *Opuntia streptacantha* cladode extracts, the authors observed an increase in the viability of cells, suggesting a potential mitogenic effect on cells [19].

The positive consequences of the anti-adipogenic effect induced by some *Opuntia ficus-indica* L. Mill. extracts are evident. Under in vivo conditions, the reduction in the number of mature adipocytes developed from pre-adipocytes and capable of accumulating large amounts of triglycerides would result in an anti-obesity effect. Nevertheless, the pro-adipogenic impact induced by some of the other extracts could also have positive consequences. In obesogenic situations, adipogenesis can be considered an interesting adaptation since small adipocytes show a more beneficial adipokine secretion profile regarding inflammation and insulin resistance. Indeed, whereas larger adipocytes are correlated with insulin resistance, dyslipidemia, high levels of inflammatory markers, and increased macrophage chemotaxis, several studies suggest that smaller adipocytes are important to avoid metabolic disorders [20, 21]. Consequently, adipocyte differentiation represents a healthier expansion of adipose tissue, acting as a preventive measure against the onset of obesity-related co-morbidities triggered by adipocyte hypertrophy [20]. Given these results, further in vivo studies using animal models are necessary to substantiate the actual effects of the analysed extracts on preventing obesity and associated co-morbidities.

### Effects of Prickly Pear Extracts on Triglyceride Content and Cell Viability in 3T3-L1 Mature Adipocytes

**Fig. 3:**
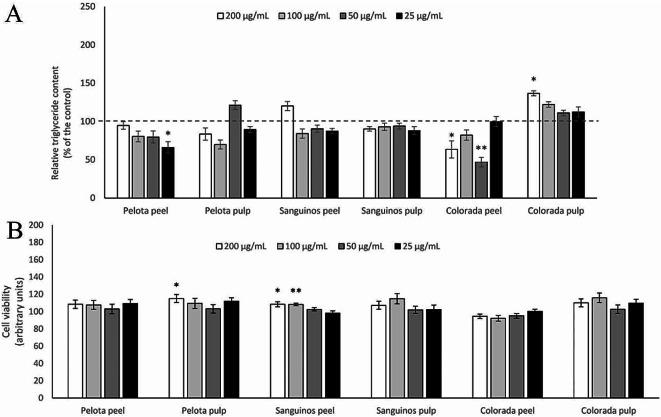
Triglyceride accumulation (**A**) and cell viability (**B**) in 3T3-L1 mature adipocytes treated for 24 h with 200, 100, 50 or 25 µg/mL extracts of Pelota, Sanguinos or Colorada *Opuntia ficus-indica* (L.) Mill. pulps and peels. Data are mean ± SEM (standard error of the mean) of three independent experiments. The intermittent line represents control values in adipocytes. Student’s *t* test was used for the analysis of comparisons between each group of treated cells and the control group (*P < 0.05; **P < 0.01)

Triglyceride accumulation was also measured in mature adipocytes treated with the prickly pear tissue extracts at the four concentrations for 24 h (Fig. 3A). Colorada peel extract, at 50 µg/mL and 200 µg/mL, reduced lipid content by -53.1% and − 36.5%, respectively. Additionally, Pelota peel extract significantly lowered triglyceride accumulation at 25 µg/mL (-33.9%). In contrast, Colorada pulp extract at 200 µg/mL significantly increased triglyceride content (36%).

Concerning cell viability, no reduction was observed, although an increase was noted in cells treated with 200 µg/mL of Pelota Pulp extract and 200 and 100 µg/mL of Sanguinos peel (Fig. 3B). Consequently, it can be stated that the observed lipid reduction in cells was not attributed to a decrease in cell viability; rather, it was a result of the triglyceride-lowering effect of the *Opuntia ficus-indica* L. Mill. extracts.

### Effects of the main Bioactive Compounds Present in Prickly pear Extracts in 3T3-L1 Maturing pre-adipocytes and Mature Adipocytes

After the initial assessment of the triglyceride-lowering effect of the three prickly pear tissue extracts, the most compelling extract was Colorada peel. This conclusion is based on the following observations: (a) it was the only one that showed a lipid-lowering effect in both pre-adipocytes and mature adipocytes, (b) this effect was induced at the lowest doses of the extract (25 and 50 µg/mL), and (c) it resulted in the highest percentages of triglyceride content reduction (-34.0% and -53.1% in pre-adipocytes and mature adipocytes, respectively). The effectiveness of this extract in reducing triglyceride accumulation in adipose cells is noteworthy. This result gains significance considering that, during the processing of *Opuntia*-based fruit beverages a substantial volume of waste and by-products is generated, primarily from the fruit peels. Indeed, there is currently a significant need for developing new strategies in managing agricultural food processing wastes and residues. Thus, the recovery of high-added value compounds from *Opuntia* wastes and by-products aligns with the objectives of the circular economy.

**Fig. 4:**
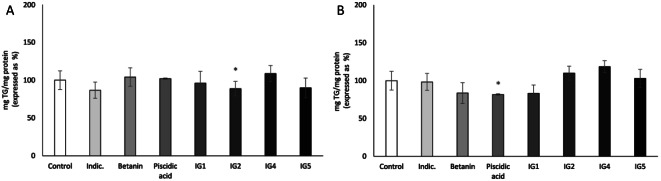
Triglyceride accumulation in 3T3-L1 maturing pre-adipocytes treated from day 0 to day 8 (**A**) and mature adipocytes treated for 24 h (**B**) with the compounds present in Colorada peel extract (50 µg/mL). Data are mean ± SEM (standard error of the mean) of three independent experiments. Student’s *t* test was used for the analysis of comparisons between each group of treated cells and the control group (*P < 0.05). Indic.: indicaxanthin; IG1: isorhamnetin glycosyl- rhamnosyl-rhamnoside; IG2: isorhamnetin glucosyl-rhamnosyl-pentoside; IG4: isorhamnetin glucosyl-pentoside; IG5: isorhamnetin glucosyl-rhamnoside

Based on this rationale, the Colorada peel extract was selected to address the second phase of the present study, which aims to determine the role of the main bioactive compounds, betalains and phenolic compounds, present in Colorada peel at 50 µg/mL, on the reduction in triglyceride accumulation in both, maturing pre-adipocytes and mature adipocytes. To accomplish this objective, maturing pre-adipocytes and mature adipocytes underwent treatment with the concentrations of the compounds corresponding to those found in Colorada peel at 50 µg/mL (Fig. 4).

When individual compounds were tested in maturing pre-adipocytes, only IG2 showed a significant reduction in lipid accumulation, specifically at 21%. Several studies have focused on the delipidating effect of isorhamnetin in cultured adipocytes, demonstrating its ability to inhibit adipogenesis and reduce lipid accumulation in cells [22–24]. However, the observed effect of IG2 was significantly lower (*p* < 0.05) than the effect produced by the Colorada peel extract. These results suggest that, while IG2 could be the primary contributor to the effect induced by the mentioned extract, the overall effect could be attributed to the additive effects of each compound individually, even in instances where statistical significance was not attained.

On the other hand, in mature adipocytes only piscidic acid proved effective in reducing triglyceride accumulation by 18% (Fig. 4B). To date, no data have been reported concerning the anti-adipogenic or delipidating activities of this phenolic compound. As in the case of pre-adipocytes, the effect of piscidic acid was lower than that induced by the Colorada peel whole extract, suggesting that the additive effects of the main bioactive compounds present in the extract explain its overall impact. With regard to the anti-adipogenic effect of phenolic acids, Aranaz et al. [25] reported that 8-day treatment of 3T3-L1 preadipocytes with ellagic, ferulic, gallic, p-coumaric and vanillic acids at 20 µM reduced *Pparγ* gene expression, although only p-coumaric acid was able to inhibit also *C/EBPα* gene expression. [25]. In the case of betalains, Chyau et al. [14] observed that betanin hindered adipogenesis by decreasing *C/ebpα* and *Srebp-1c* gene expression [14].

Therefore, it can be stated that the lipid-lowering effect of *Opuntia ficus-indica* is related to the different bioactive compound composition. In addition, in spite of the promising results obtained, further studies are necessary to enhance our understanding of the amount of the bioactive compounds found in Pelota, Sanguinos and Colorada that can actually reach the bloodstream, and, consequently, the adipose tissue following the oral administration of the extracts. In this regard, it has been reported that after an in vitro digestion, betalains were stable enough to reach the intestinal phase, with indicaxanthin exhibiting higher bioaccessibility compared to betanin [9]. Furthermore, some studies have observed that indicaxanthin and betanin are not metabolised in the stomach or the liver [26]. On the other hand, it has been noted that phenolic compounds are highly abundant in prickly pear peels, and they exhibit high stability and bioaccesibility during digestion [9]. With regard to the IGs, it has been stated that the plasma stability of IG´s is better compared to the aglycone forms, which are less effectively retained in the circulatory system [27]. Moreover, further studies are needed to investigate the phase II and microbial metabolites derived forms from the bioactive compounds present in the *Opuntia* extracts, as well as to define whether these metabolites are active molecules with promising lipid-lowering effects.

## Conclusions

It can be concluded that the three varieties of *Opuntia ficus-indica* L. Mill. (Mexican Pelota; Spanish Sanguinos and Colorada) display different compositions in betalains and phenolic compounds, and they show varying effectiveness in reducing the lipid content in both 3T3-L1 maturing pre-adipocytes and mature adipocytes. Among the six *Opuntia ficus-indica* L. Mill. extracts analysed, Colorada peel extract was selected as the most interesting one. It demonstrated effectiveness in both pre-adipocytes and mature adipocytes, inducing the strongest delipidating effects at the lowest tested doses. With these results, further research devoted to analyse the in vivo effects of prickly pear extracts in the prevention and treatment of obesity is needed. Furthermore, a better stablishment of the digestion stability and bioavailability of the bioactive compounds present in the extract should be interesting, as well as the potential bioactivity of their derived metabolites. In addition to the fact that the use of prickly pear as a source of bioactive compounds appear promising, it is in good accordance with the objectives of the circular economy.

### Electronic Supplementary Material

Below is the link to the electronic supplementary material.

Supplementary Material 1

Supplementary Material 2

## Data Availability

The experimental data are included in the manuscript and is available from the corresponding author upon request.

## Abbreviations

IG: Isorhamnetin glycosides
WHO: World health organization
