# Supplementary figures and images for: In Vitro Screening and Lipid-Lowering Effect of Prickly Pear (Opuntia Ficus-Indica L. Mill.) Fruit Extracts in 3T3-L1 Pre-Adipocytes and Mature Adipocytes
Source: Plant Foods Hum Nutr. 2024 Jan 11;79(1):143–50. doi: 10.1007/s11130-023-01137-8 (PMC10891207; doi:10.1007/s11130-023-01137-8)

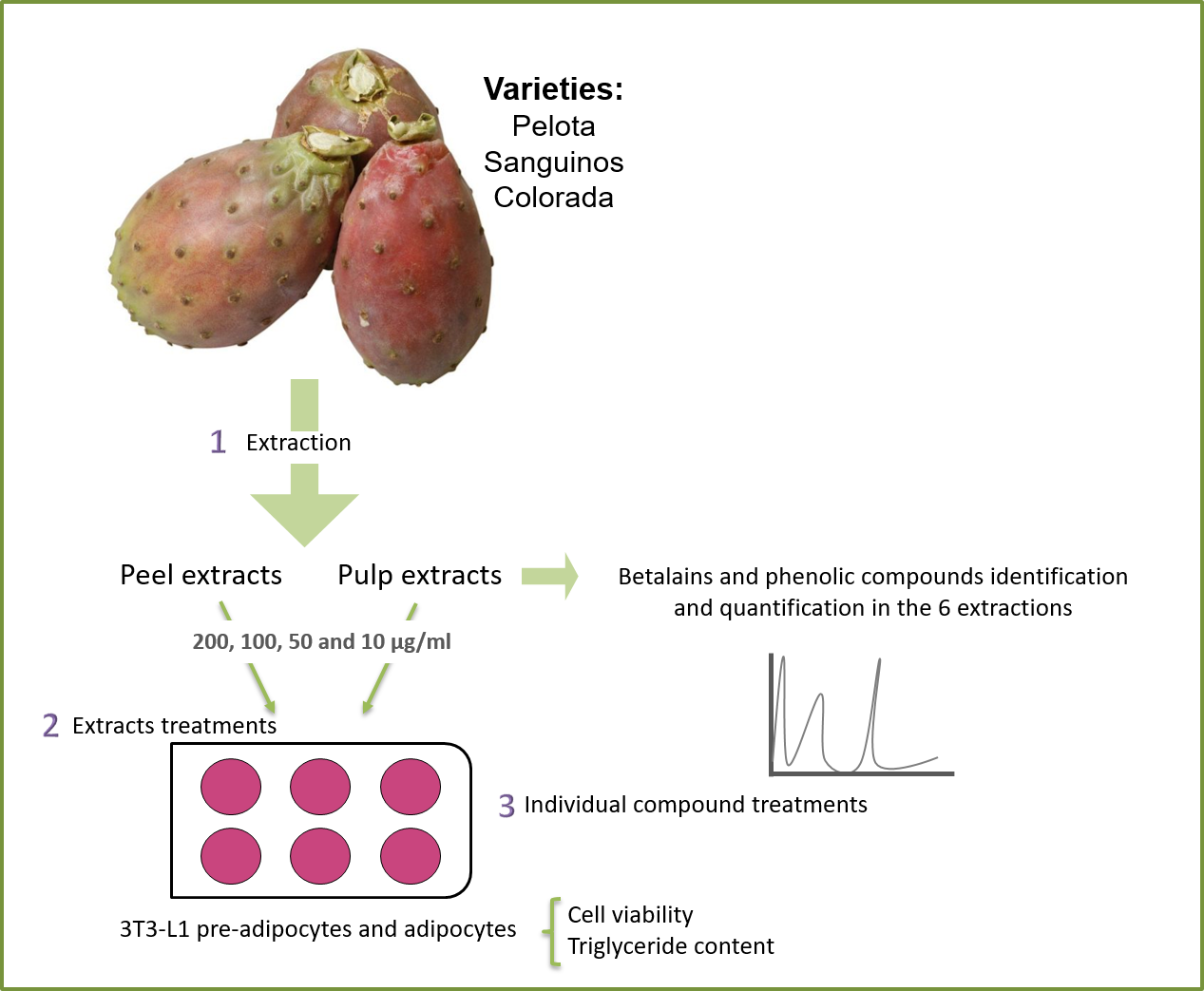

Supplement: Supplementary file 1 — Supplementary Material 1 [file 11130_2023_1137_MOESM1_ESM.png]
