# Supplementary material for: In Vitro Screening and Lipid-Lowering Effect of Prickly Pear (Opuntia Ficus-Indica L. Mill.) Fruit Extracts in 3T3-L1 Pre-Adipocytes and Mature Adipocytes
Source: Plant Foods Hum Nutr. 2024 Jan 11;79(1):143–50. doi: 10.1007/s11130-023-01137-8 (PMC10891207; doi:10.1007/s11130-023-01137-8)
Supplement: Supplementary file 2 — Supplementary Material 2 [file 11130_2023_1137_MOESM2_ESM.docx]

**Online Resource**

***In vitro* screening and lipid-lowering effect of prickly pear (*Opuntia ficus-indica L. Mill.*) fruit extracts in 3T3-L1 pre-adipocytes and mature adipocytes**

Itziar Eseberri ^1,2,3*^, Andrea Gómez-Maqueo^4^, Jenifer Trepiana^1,2,3^, Iván Gómez-López^1,3,4^, Carina Proença^5^, M. Pilar Cano^4^, Maria P. Portillo^1,2,3^

^1^ Nutrition and Obesity Group. Department Nutrition and Food Science, University of the Basque Country (UPV/EHU) and Lucio Lascaray Research Institute, Vitoria, Spain.

^2^Bioaraba Health Research Institute, Vitoria, Spain.

^3^ CIBERobn Physiopathology of Obesity and Nutrition, Institute of Health Carlos III, Madrid, Spain.

^4^ Department of Biotechnology and Microbiology of Food, Institute of Food Science Research (CIAL, CSIC-UAM), Nicolás Cabrera 9, 28049, Madrid, Spain.

^5^ LAQV, REQUIMTE, Laboratory of Applied Chemistry, Department of Chemical Sciences, Faculty of Pharmacy, University of Porto, Porto, Portugal.

**Correspondence:** Itziar Eseberri. E-mail: itziar.eseberri@ehu.eus

**Material and methods**

***Prickly pear fruits and extracts***

The prickly pear fruits used in this study were obtained from Mexican and Spanish *Opuntia ficus-indica* (L.) Mill. varieties. Spanish Orange Colorada prickly pear fruits were provided by Fasnia (Tenerife, Canary Islands, Spain; 28°2’N, 16°4’W; 446 m over sea level), Spanish red Sanguinos prickly pear fruits were purchased from Bioarchen in Archen (Murcia, Spain; 38°7’N, 1°18’W; 121 m over sea level) and purple Mexican Pelota prickly pear fruits were donated by Agroproductores La Flor de Villanueva in San Sebastián Villanueva Acatzingo (Puebla, Mexico; 19°1’N, 97°4’W; 2150 m over sea level). Fruits were washed and selected according to uniform maturity, size, and no defects and then separated into peels and pulps. Tissues were cut into small pieces (20 x 20 mm), vacuum-sealed in polyethylene bags, and frozen with liquid nitrogen. Samples were freeze-dried for 5 days at -45°C and 1.3 x 10-3 MPa (LyoBeta 15, Azbil Telstar, S.L., Terrasa, Spain), pulverized (Grindomix GM200, Retsch, Germany) to a fine particle size (< 2 mm) and seeds were removed. Aqueous prickly pear extracts were obtained from freeze-dried tissues by extracting with methanol:water (1:1, v:v) several times and methanol to obtain the extracts rich in betalains and phenolic compounds [1]. Aliquots of each extract were freeze-dried and stored at - 20°C until their use for HPLC analysis and cellular experiments with 3T3-L1 maturing pre-adipocytes and mature adipocytes.

***Bioactive compound quantification by high-performance liquid chromatography***

The identification of betalains and phenolic compounds in prickly pear fruit extracts was performed by high-performance liquid chromatography using a 1200 Series Agilent HPLC System (Agilent Technologies, Santa Clara, CA, USA) with a reverse-phase C18 column (Zorbax SB-C18, 250 x 4.6 mm i.d., S-5 µm; Agilent) at 25°C [1,2]. The determination of each compound was given according to their retention times, comparing UV/Vis and mass spectral data to those of purified, semi-synthesized or commercial standards. Specifically, quantification of indicaxanthin, betanin, piscidic acid, and isorhamnetin glycosides was carried out with the calibration curves of the corresponding isolated and semi-synthetized standards, by calibration curves for each of the standards (five points in a range of 0-300 µg/mL). The compound amount is expressed as µg/g dry weight. The obtained chromatograms are included in the following figure (Online Resource-Figure 1).

**
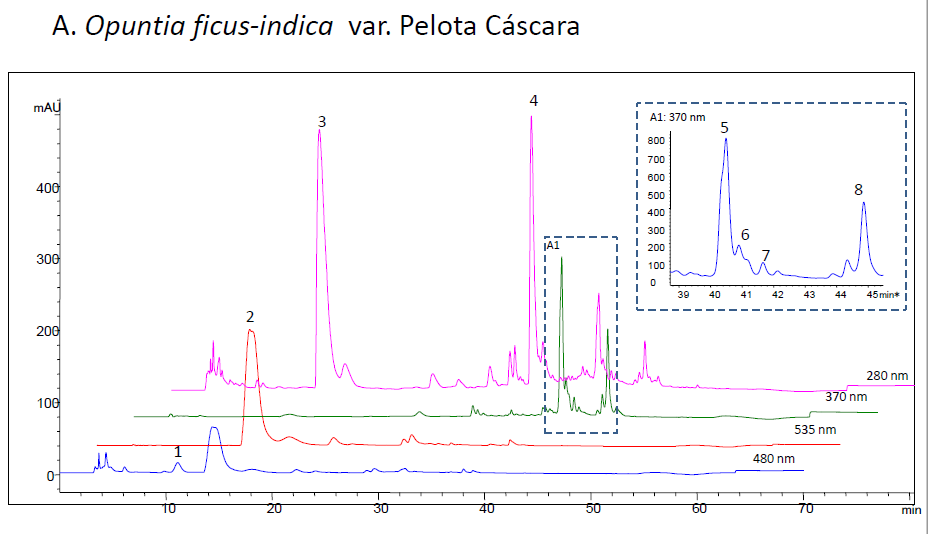
**

A


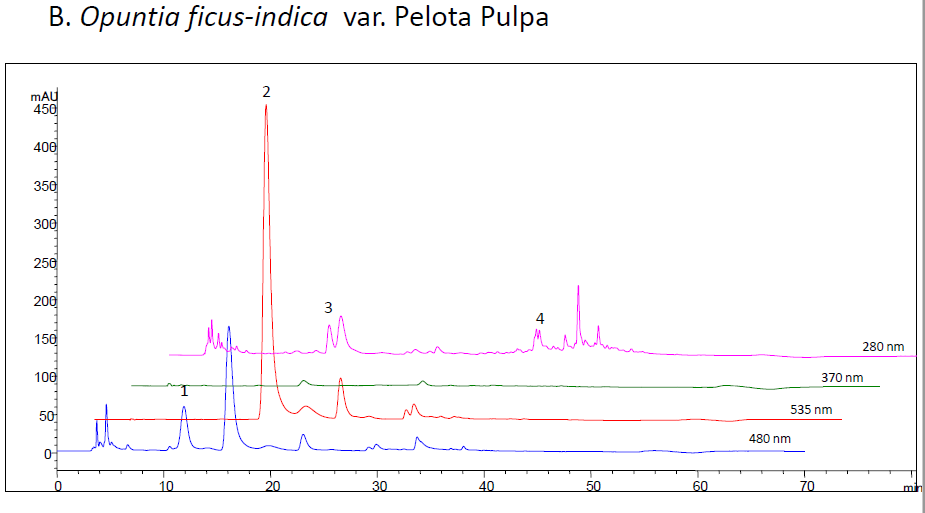


B


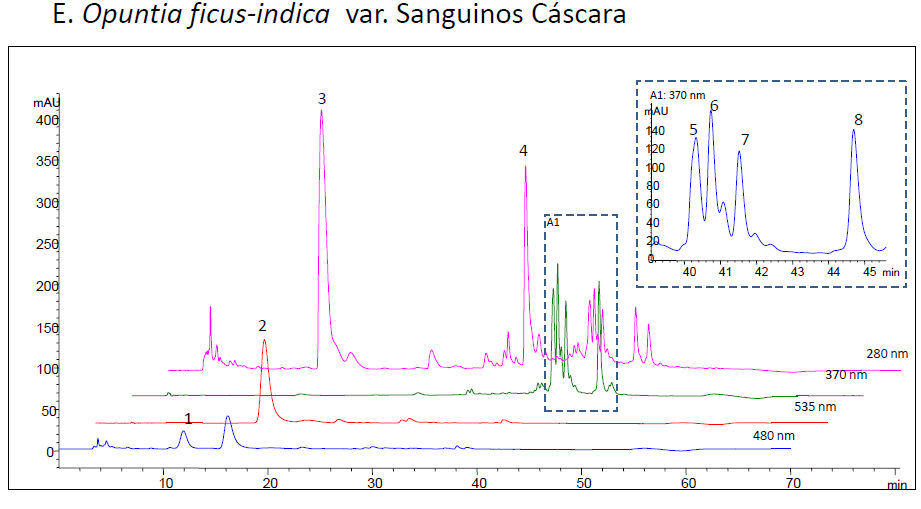


C


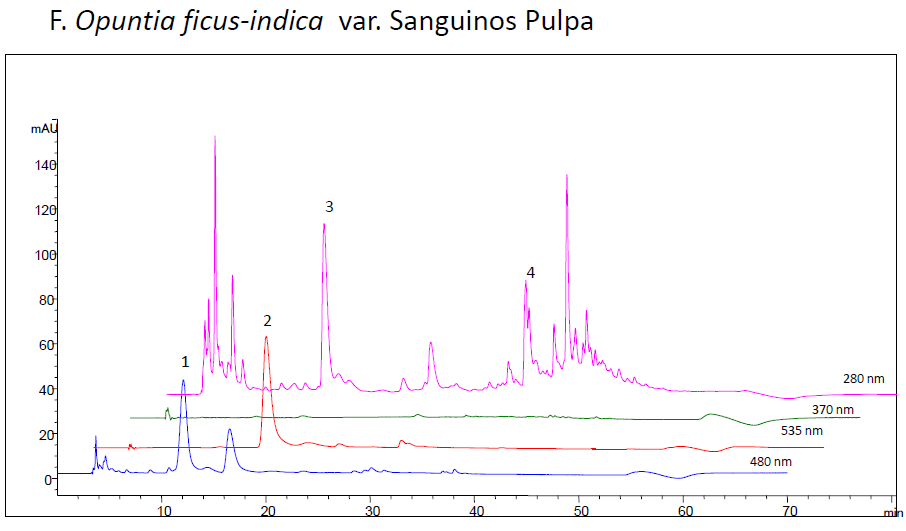


D


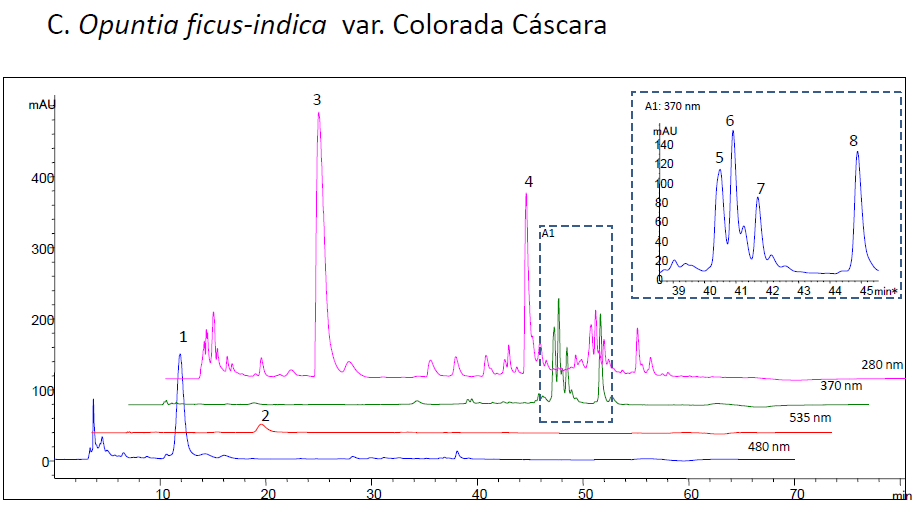


E


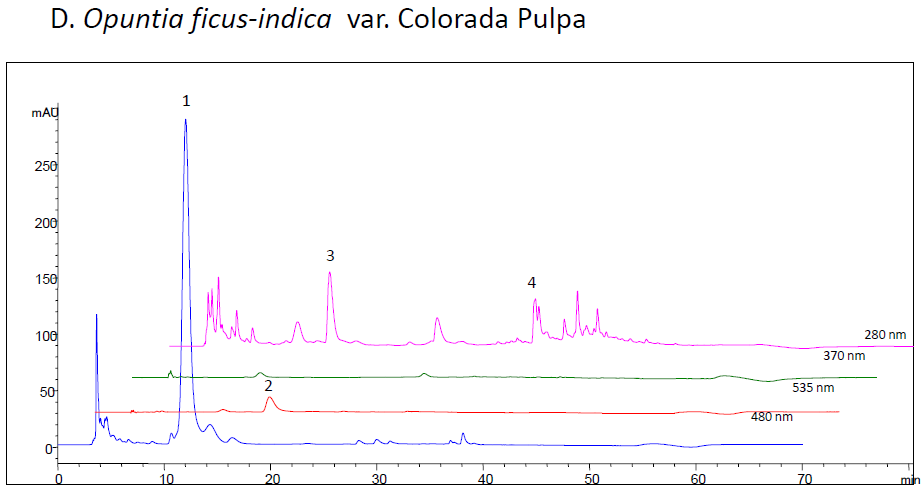


F

**Online Resource-Figure 1**. Quantification of betalains and phenolic compounds in Pelota peel (A), Pelota pulp (B), Sanguinos peel (C), Sanguinos pulp (D), Colorada peel (E), Colorada pulp (F) prickly pear fruit extracts performed by high performance liquid chromatography. ^1^indicaxanthin; ^2^betanin; ^3^piscidic acid; ^4^hydroxybenzoic acid glycoside; ^5^isorhamnetin glucosyl rhamnosyl rhamnoside (IG 1); ^6^isorhamnetin glucosyl rhamnosyl pentoside (IG 2); ^7^isorhamnetin glucosyl pentoside (IG 4); ^8^isorhamnetin glucosyl rhamnoside (IG 5).

***Isolation and semi-synthesis of betalains and phenolic compounds present in the tissues of Opuntia ficus-indica***

Beetroot was used to obtain an extract with a high amount of betanins, which was purified in a Sephadex L20 resin. In the case of indicaxanthin, it was semi-synthesized from purified betalain by using ammonia to increase pH and get betalamic acid, which reacts with proline to obtain the compound. Extracts of *Opuntia ficus-indica* peels were used to isolate piscidic acid by semi-preparative high-performance liquid chromatography (HPLC). In Sergio Serna-Saldivar’s laboratory in Centro de Biotecnología FEMSA (Mexico), researchers extract isorhamnetin glycosides from Opuntia cladodes, which have been provided to us for this research.

***Cell experimental design***

3T3-L1 preadipocytes purchased from American Type Culture Collection (Manassas, VA, USA) were seeded in 6-well plates and incubated in DMEM supplemented with 10% fetal bovine serum (FBS) and 5% penicillin/streptomycin (PS; 10 000 U/mL) under optimal growing conditions (37ºC and 5% CO_2_-humidified atmosphere). Post-confluent pre-adipocytes were stimulated to differentiate using the same previous incubation media containing insulin (10 µg/mL), isobutylmethylxanthine (0.5 mM), and dexamethasone (1 µM) for 48 hours. After 2 days, cells were incubated with DMEM/FBS/PS medium containing 10 µg/mL insulin for 48 hours, and from day 4 onwards, insulin concentration was reduced to 2 µg/mL in the incubation media, which was replaced every two days. Cells were harvested on day 8 of differentiation for maturing pre-adipocyte treatments, and day 12 in the case of mature adipocyte treatments, where over 90% of cells have visible lipid droplets.

***Cell treatment***

Maturing pre-adipocytes were incubated with prickly pear (*Opuntia ficus-indica* L. Mill.) extracts or each isolated compound during the adipogenic phase, from day 0 to day 8 of differentiation by replacing the treatment media every 48 hours. In the case of mature adipocytes, cells were treated on day 12 of differentiation for 24 hours with prickly pear (*Opuntia ficus-indica* L. Mill.) extracts or each isolated compound. To obtain the tested concentrations of extracts and isolated compounds, freeze-dried samples were dissolved in water (peel and pulp extracts, indicaxanthin and betanin) or methanol (piscidic acid and isorhamnetin glycosides), and added to cells in a final concentration of 0.1% of the incubation media. Control methanol cells were treated with the same amount of the solvent (0.1% of the incubation media). At the end of the experimental period, the incubation media was removed and cells were used for triglyceride and protein determination.

In both cases, two independent experiments were carried out; in the first one, cells were treated with the peel or pulp extracts of each of the three prickly pear varieties at 200, 100, 50 and 25 µg/mL. The range of concentrations used was consistent with those in many other studies regarding the effect of phytochemicals on triglyceride accumulation in 3T3-L1 pre-adipocytes and mature adipocytes. In the second experiment, focused on the extract selected in the first experiment, cells were treated with the amounts of the isolated compounds, indicaxantin, betanin, piscidic acid, isorhamnetin glycosyl- rhamnosyl-rhamnoside (IG1), isorhamnetin glucosyl-rhamnosyl-pentoside (IG2), isorhamnetin glucosyl-pentoside (IG4) and isorhamnetin glucosyl-rhamnoside (IG5), present in cell culture medium when this selected extract induced triglyceride-lowering effects (Online Resource-Table 1). To calculate these amounts, the composition of extracts shown in Table 1 of the Results section of the article was used. Each experiment was performed three times.

**Online Resource-Table 1.** Amount of each compound in the incubation media of treated cells

| **Bioactive compound** | **Compound quantity in the incubation media (µg/mL)** |
| --- | --- |
| Indicaxantin | 0.0225 |
| Betanin | 0.0045 |
| Piscidic acid | 2.5495 |
| IG1 | 0.0110 |
| IG2 | 0.0105 |
| IG4 | 0.0060 |
| IG5 | 0.0160 |

***Cell viability assay***

Cells were seeded and incubated in 96-well plates at the same incubation conditions as described before and treated with all prickly pear (*Opuntia ficus-indica* L. Mill.) peel or pulp aqueous extracts at 200, 100, 50 and 25 µg/mL. For cell viability evaluation, it was performed the violet coloration of living cells as previously described [3]. For cell coloration, the incubation medium was removed and cells were washed with sterile phosphate buffer saline (PBS) 10 mM. After that, cells were fixed with formaldehyde (3.7%), and after a double PBS whasing, stained in the dark with crystal violet (0.25%) for 20 minutes. Once the wells were rinsed to remove the excess of dye, the formed crystals were dissolved in acetic acid at 33% and the absorbance (590 nm) was measured with an iMark microplate reader (Bio-Rad, Hercules, CA, USA). Results are expressed in arbitrary units.

***Measurement of triglyceride and protein content in 3T3-L1 maturing pre-adipocytes and mature adipocytes***

At the end of the experimental period, the incubation medium was removed and cells were washed with PBS 10 mM. Subsequently, a harvest buffer (Tris-HCl pH 7.4, 150 mM NaCl, 1 mM EDTA, 100 mM phenylmethylsulfonyl fluoride) was used to collect cells using a cell scraper. Then, cells were exposed to a sonication by a Branson Digital Sonifier SFX 550 (Emerson Electric Co, St Louis, MO, USA) before the measurement of triglycerides using a commercial kit (Spinreact, Girona, Spain) and the protein content of each well following the Bradford method [4]. Results are expressed as mg of triglycerides per mg of protein.

***Statistical analysis***

Results are presented as mean ± SEM (standard error of the mean) of three independent experiments carried out in sextuplicates. Statistical analysis was performed using SPSS 26.0 (SPSS Inc. Chicago, IL, USA). Data from cells treated with each extract was compared to control cells by using Student’s *t* test. Statistical significance was determined at *p* < 0.05.

# References

1.Gomez-Maqueo A, Antunes-Ricardo M, Welti-Chanes J, Cano MP (2020) Digestive Stability and Bioaccessibility of Antioxidants in Prickly Pear Fruits from the Canary Islands: Healthy Foods and Ingredients. Antioxidants (Basel) 9:164. doi: 10.3390/antiox9020164.

2.García-Cayuela T, Gómez-Maqueo A, Guajardo-Flores D, Welti-Chanes J, Cano MP (2019) Characterization and quantification of individual betalain and phenolic compounds in Mexican and Spanish prickly pear (Opuntia ficus-indica L. Mill) tissues: A comparative study. Journal of Food Composition and Analysis 76:1-13. doi: <https://doi.org/10.1016/j.jfca.2018.11.002>

3.Gillies RJ, Didier N, Denton M (1986) Determination of cell number in monolayer cultures. Anal Biochem 159:109-113. doi: <http://dx.doi.org/10.1016/0003-2697(86)90314-3>

4.Bradford MM (1976) A rapid and sensitive method for the quantitation of microgram quantities of protein utilizing the principle of protein-dye binding. Anal Biochem 72:248-254
